# Viability of Neural Cells on 3D Printed Graphene Bioelectronics

**DOI:** 10.3390/bios9040112

**Published:** 2019-09-20

**Authors:** Jingshuai Guo, Amir Ehsan Niaraki Asli, Kelli R. Williams, Pei Lun Lai, Xinwei Wang, Reza Montazami, Nicole N. Hashemi

**Affiliations:** 1 Department of Mechanical Engineering, Iowa State University, Ames, IA 50011, USA; jguo1@iastate.edu (J.G.); niaraki@iastate.edu (A.E.N.A.); kellij2@iastate.edu (K.R.W.); issaclun@iastate.edu (P.L.L.); xwang3@iastate.edu (X.W.); reza@iastate.edu (R.M.); 2 Department of Biomedical Engineering, Iowa State University, Ames, IA 50011, USA

**Keywords:** Parkinson’s Disease, mechanically exfoliated graphene, neuronal cells, electrical conductivity, biosensor

## Abstract

Parkinson’s disease (PD) is the second most common neurodegenerative disease in the United States after Alzheimer’s disease (AD). To help understand the electrophysiology of these diseases, N27 neuronal cells have been used as an in vitro model. In this study, a flexible graphene-based biosensor design is presented. Biocompatible graphene was manufactured using a liquid-phase exfoliation method and bovine serum albumin (BSA) for further exfoliation. Raman spectroscopy results indicated that the graphene produced was indeed few-layer graphene (FLG) with ${\left(I_{D}/I_{G}\right)}_{Graphene}=$ 0.11. Inkjet printing of this few-layer graphene ink onto Kapton polyimide (PI) followed by characterization via scanning electron microscopy (SEM) showed an average width of ≈868 µm with a normal thickness of ≈5.20 µm. Neuronal cells were placed on a thermally annealed 3D printed graphene chip. A live–dead cell assay was performed to prove the biosensor biocompatibility. A cell viability of approximately 80% was observed over 96 h, which indicates that annealed graphene on Kapton PI substrate could be used as a neuronal cell biosensor. This research will help us move forward with the study of N27 cell electrophysiology and electrical signaling.

## 1. Introduction

Parkinson’s disease (PD) is a neuronal disease that is caused by the death of dopaminergic neurons in the substantia nigra pars compacta region of the brain [1]. It is currently the second most common neurodegenerative disease in the United States after Alzheimer’s disease (AD) [1,2]. Because of their dopaminergic properties [1], rat dopaminergic N27 cells have been widely used in in vitro models for PD studies [1,3]. They have also been used in studies seeking to understand problems such as neurotoxicity, oxidative stress, and histone deacetylase (HDAC) and other molecular pathways [4,5,6]. While there have been studies on synaptophysin signaling in N27 cells, the electrophysiological effects of graphene on N27 cells are currently unknown. Since a study on the electrophysiology of N27 cells is necessary to further understand PD and other neurodegenerative diseases, graphene may be helpful to better understand neurodegenerative diseases when employed as a biosensor.

Graphene is among the most widely used materials in the field of material science [7,8]. Graphene has a two-dimensional honeycomb nanostructure with a one-atom-thick planar sheet of sp^2^-bonded carbon atoms [8,9,10]. Graphene has a large theoretical specific surface area of 2630 m^2^ g^−1^ [11,12], intrinsic mobility of 200,000 cm^2^v^−1^s^−1^ [13], and a superior thermal conductivity of approximately 5000 Wm^−1^K^−1^ [14]. Since it also has excellent electrical conductivity, it lends itself to many real-world applications and provides an ideal foundation for bioelectronics and biosensing [15,16].

Although there are currently many methods used to produce graphene, the end products are not biocompatible [17,18,19]. For example, popular graphene solutions such as dimethylformamide (DMF) and N-methylpyrrolidone (NMP) solutions are toxic and often result in low-concentration graphene solutions [20,21]. Therefore, it is necessary to develop a cell-friendly approach for large-scale production of biocompatible graphene. To address this problem, research was conducted on the synthesis of graphene-based solutions via oxidation and chemical vapor deposition (CVD) with the intent of improving these graphene fabrication methods [22,23,24,25]. However, there is evidence showing that the Hummer’s method creates single carbon atom defects along with nano-sized cracks [26]. CVD works well for producing large, continuous films of graphene, but this method has been shown to result in numerous surface voids and defects [27], and its application has been only partly successful [28]. As the research of biocompatible graphene and its implementation in practical applications such as printing graphene have continued to grow, very few studies have been able to produce satisfying results [21]. Fortunately, the method of direct liquid-phase exfoliation (LPE) of graphite into graphene has been reported as an attractive approach for inkjet printing and cell-based studies.

There have been many previous studies on constructing biosensors using graphene-based materials [29]. One such example involved using graphene oxide (GO) to examine cellular DNA via the creation of graphene quantum dots (GQDs) [30]. The biosensors in another study primarily focused on the transfer of electrons and used CV to image cancer stem cells [31]. Also, while [Fe(CN)_6_]^3−/4−^ and [Ru(NH_3_)_6_]^3+/2+^ redox peaks have been measured, a study on the bioelectrical signaling of neuronal cells using graphene-based sensors is lacking [30,31,32,33]. One advantage of using graphene in a biosensor study is that this material has no effect on mitochondrial membrane potential (MMP), mitochondrial morphology, or cell stress status [34]. Since graphene appears to have a bright future in biochemical and biomedical applications, engineers are encouraged to continue research studies on this material [34,35,36,37].

In this study, we describe a prototype graphene biosensor to sense electrical signaling in N27 cells. The biosensor was produced with an inkjet printer to create conductive, biocompatible, and defect-free graphene. The graphene was exfoliated from graphite using the LPE method. This design presents a facile technique that can be used to manufacture biosensors for a variety of applications. In a previous study, bovine serum albumin (BSA) was used to exfoliate graphene in an aqueous state and resulted in graphene that exhibited strong biocompatibility. Since this technique was easily implemented, it resulted suitable for large-scale graphene production and, hence, was adopted in this study. The graphene and printed graphene chips were characterized using Raman spectroscopy, Atomic force microscopy (AFM), and scanning electron microscopy (SEM). To characterize cell viability within the biosensor, multiple live–dead cell assays were performed.

## 2. Materials and Experimental Section

### 2.1. Materials and Equipment

The materials and equipment used in this study were: graphite (Synthetic powder, <20 µm, Sigma-Aldrich, St. Louis, MO, USA); Albumin, from bovine serum (bovine albumin* BSA, ≥98% agarose gel electrophoresis, lyophilized powder, Sigma-Aldrich, St. Louis, MO, USA); poly(sodium 4-styrenesulfonate) (PSS, Mw ~1,000,000 powder, Sigma-Aldrich, St. Louis, MO, USA); poly(ethyleneimine), (PEI) solution (50% *w*/*v*, Sigma-Aldrich, St. Louis, MO, USA); sodium chloride (NaCl) purchased from Sigma-Aldrich, St. Louis, MO, USA; Kapton polyimide (PI) (thickness: 0.008 mm, 100 × 100 mm, Sigma-Aldrich, St. Louis, MO, USA). RPMI medium 1640 (1X) (Ref#: 11875-093, 500 mL), L-glutamine 200 mM(100X) (Ref#: 25030-081, 100 mL), pen/strep solution (penicillin 10,000 U mL^−1^/streptomycin 10,000 μg mL^−1^, Ref#: 15140-122, 100 mL) was purchased from Gibco Life Technologies. Fetal bovine serum (FBS) (Qualified One Shot™, Ref#: A31606-01, 50 mL) was purchased from ThermoFisher Scientific, Waltham, MA. Polyurethane ether tubes (I.D.:0.063″, Wall:0.031″, O.D.:0.125″, Part Number: 2100070-100) were purchased from Superthane^®^, 3 mL Luer-Lok syringes were purchased from Allegro Medical. Inc. (Bolingbrook, IL, USA) Six-well cell culture clusters (Lot# 23314037) were purchased from Costar^®^. A GenieTouch™, syringe pump was purchased from Kent Scientific Corporation. A 4-axis CNC USB controller Mk3/4 for mini CNC mill was purchased and controlled by a PlanetCNC^®^ (Ljubljana, Slovenia). A sinometer digital multimeter (MS8261) was used to measure print conductivity. A JEOL FESM JCM-6000 scanning electron microscope and a Zeiss Axio Observer Z1 inverted microscope were used for SEM imaging of the graphene prints and the live–dead cell assays, respectively. A Raman spectrometer (Voyage, B&W Tek, Inc., Newark, DE, USA) with a CW laser (Excelsior-532-150-CDRH, Spectra-Physics) was used for Raman spectroscopy measurements. A PerkinElmer UV–Vis spectrophotometer (Lambda 750) at λ = 660 nm at room temperature was used to provide absorption spectra and consequently the concentration of graphene ink.

### 2.2. Preparation of Graphene

**Figure 1:**
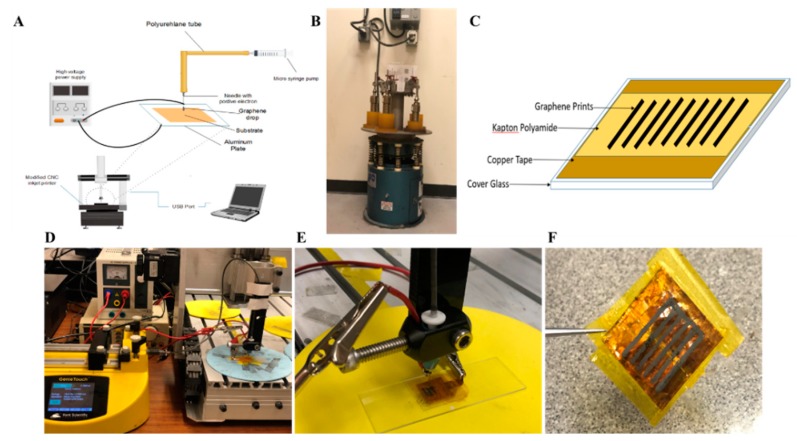
Schematic of the drop-on-demand graphene printing method. (**A**) An abridged general view of the printer setup. (**B**) A graphene solution was mixed using a Vibro-Energy mill (shaker mill) for 90 h at 300 rpm. (**C**) Schematic of chip setup. (**D**) Equipment setup during printing. A syringe pump was used along with an inkjet printer to deposit ink at a rate of 7 µL/s to ensure a constant flow rate throughout the syringe. (**E**) Needle and substrate setup. A 3 kV potential difference was introduced between the substrate and needle for the purpose of affixing ink onto the substrate. A cover glass was placed on a regular microscope slide for stability and support. (**F**). Printed chip end result of 3D printed graphene.

**Figure 2:**
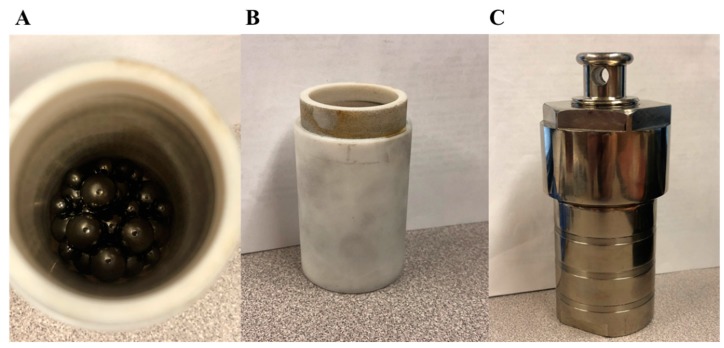
Graphene wet-ball milling container setup. (**A**) Twenty steel balls with a diameter of 8.7 mm (11/32″) and 10 steel balls with a diameter of 4.5 mm (3/16″) were added. (**B**,**C**) Container assembly for shaker mill.

The graphene solution used in this study was produced using the liquid exfoliation method. Wet-ball milling was used with both the Vibrio-Energy shaker mill [38] and a kitchen blender for further exfoliation [8]. The preparation of graphene, ink formulation and finally inkjet printing of the produced conductive ink is illustrated in Figure 1.The shear tension created by the steel balls helped further the exfoliation of graphene crystallites and led to the fabrication of high-quality FLG [38,39,40]. When using the Vibro-Energy (shaker) mill, 650 mg of graphite was mixed with 60 mg of BSA and was well dispersed in 35 mL of DI-H_2_O. The Vibro-Energy mill (shaker mill) was run for 90 h at 300 rpm (Figure 1B). Twenty steel balls with a diameter of 8.7 mm (11/32″) and 10 steel balls with a diameter of 4.5 mm (3/16″) were added to all 5 containers (Figure 2A). A standard kitchen blender was run for 1 h at a speed of 16,761 rpm. Twenty grams of graphite was mixed with 605 mg of BSA well dispersed in 100 mL of DI-H_2_O. Both graphene solutions were kept at rest for 24 h, allowing any remnant of non-dispersed graphite particles to sink to the bottom of the containers. Finally, in order to ensure repeatability of the formulated ink, the samples were collected from the top 80% of the solution, and the concentration of graphene was measured through UV–Vis spectroscopy. The resultant concentration resulted to be $C\approx 5.1\;{\mathrm{mg}\;\mathrm{mL}}^{-1}$.

### 2.3. Substrate Preparation

Because of its great thermal resistance, Kapton PI was chosen as the polymer substrate. It can withstand temperatures from −269 to 400 °C. Due to the hydrophilicity and negative charge of the FLG ink, it was necessary to perform a hydrophilicity treatment on the Kapton PI substrate. To treat Kapton PI, PI was first washed in acetone. The substrate was next washed with PSS (3.5 mg/mL), submerged in a solution of 50 mL DI-H_2_O and NaCL (0.5 mol L^−1^), and finally submerged into a PEI solution (30 mg/mL) of DI-H_2_O and an NaCl solution (0.5 mol L^−1^) for additional 20 min. The substrate was then allowed to air-dry for 12 h.

### 2.4. Inkjet Printing Procedure

The graphene ink was placed in a 5 mL syringe, and a 10 cm-long polyurethane ether tube was attached to this syringe. The graphene ink was injected through a hypodermic needle with an inner diameter of 300 µm. The syringe was fixed, and the graphene ink was injected via the syringe pump at a rate of 3 uL s^−1^. The ink was injected onto the PI substrate, which was fixed on a 22 × 22 mm glass cover chip. The chip was covered with two pieces of copper tape, with a 0.50 mm gap between the two pieces (Figure 1C). A 3 kV potential difference was applied between the needle and the substrate, with the needle position being controlled by the CNC mini mill and its associated software. Prints were manufactured with a space of 800 µm between each print, and a total of five graphene lines were printed on one chip. Figure 1F shows the final printing result after annealing. A total of six graphene chips were made during each printing session.

### 2.5. Post-Processing and Conductivity Testing

Post-processing was performed for the purpose of improving the electrical conductivity and the stability of the printed graphene [41,42]. A set temperature of 280 °C was used to anneal the inkjet-printed graphene. After 30 min of annealing, the graphene chips were gently placed in a clear six-well plate. All chips required sterilization before introducing the cells. This allowed the cells to grow and increased the chances of their survival. A digital multimeter was used for conductivity testing, and the resistance of each line was measured every 3 mm across the gap created on the chip. The width of each line was then measured using a SEM and found to be 868±20 μm; the height of each line measured by SEM was 4±1 μm. The conductivity of each line could then be calculated from Equation (1), where *R* is the resistance of a printed line, *l* is its measured length, *w* is its width, and *σ* is the conductivity [43,44].

(1) $$R=\frac{l}{wt\sigma }$$

**Figure 3:**
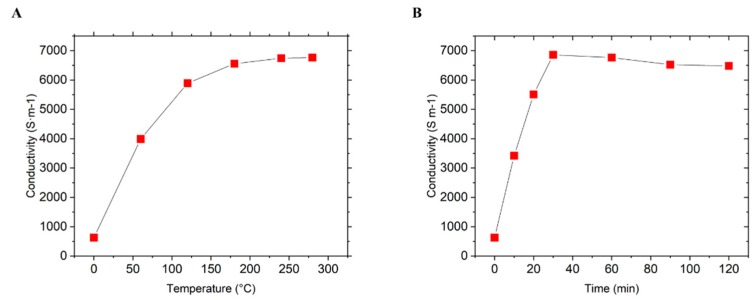
Conductivity measurements of printed graphene prints after treatment. (**A**) Conductivity changes dependent on the temperature, checked every 30 min. (**B**) Conductivity changes dependent on time at the same temperature of 280 °C.

Based on Equation (1), the conductivity measurements were performed by serval trails with different annealing temperatures and different time periods. Figure 3 shows the conductivity changes along annealing time and temperature change.

### 2.6. Raman Spectroscopy

**Figure 4:**
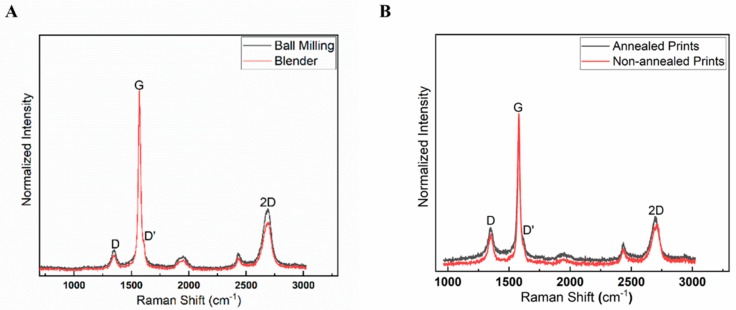
Raman spectra plot of both ball milling and blundered graphene under 532 nm laser. (**A**) Raman results for both ball milling and blender graphene samples on a Si/SiO_2_ substrate. (**B**) Raman results for after annealed prints and non-annealed prints on Kapton tape.

The samples obtained from the shaker mill and kitchen blender had similar cartelization when Raman spectroscopy was performed (Figure 4A). A thin film of graphene sample was prepared to determine the Raman spectra. A drop of the graphene sample with a diameter of approximately 10 mm was placed on top of a Si/SiO_2_ substrate and air-dried. A Raman spectrometer with a CW laser provided a laser beam at wavelength 532 nm to the graphene on the Si/SiO_2_ sample. Figure 4A depicts the Raman results for the graphene sample, on which five points were acquired by the Raman spectra. Figure 4B depicts the Raman results for the printed graphene lines on the Kapton substrate with comparisons of annealed graphene prints and non-annealed graphene prints.

### 2.7. Scanning Electron Microsopy and Atomic Force Microscopy

**Figure 5:**
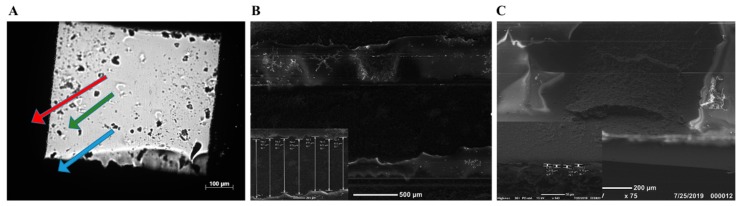
Microscope image of printing results. (**A**) An area of prints under the microscope. The red, blue, and green arrows indicate graphene, copper tape, and polyimide (PI), respectively, after the annealing process. (**B**,**C**) SEM images of printed graphene on PI using 2–5 kV accelerated voltage. (**B**) is a top-view image taken using SEM after annealing the prints with an average width of ≈868 µm. (**C**) shows an image of a cross section with an average depth of 5.20 µm.

**Figure 6:**
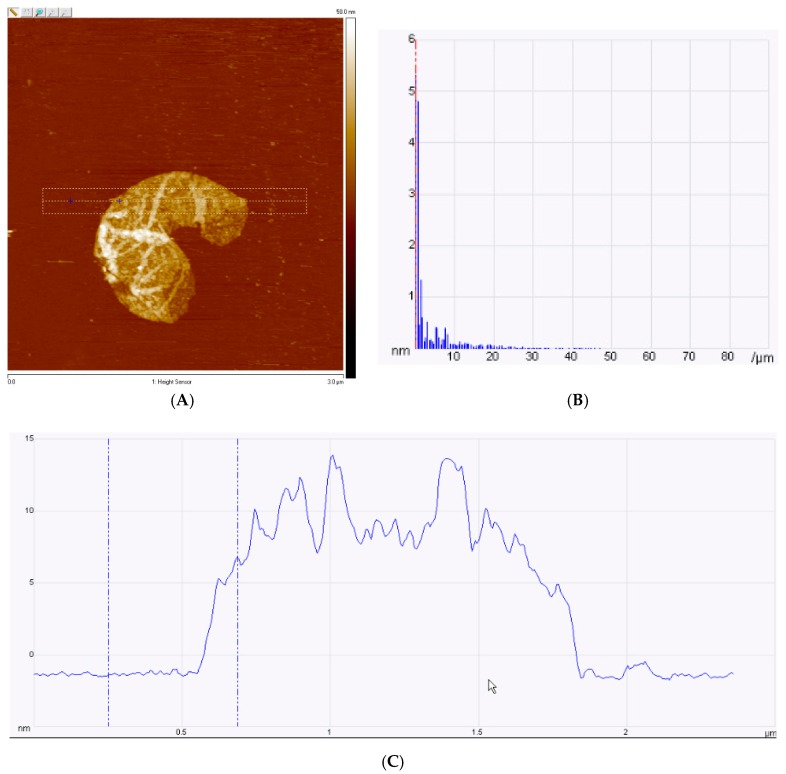
Atomic force microscopy (AFM) of graphene. (**A**) Graphene flake, (**B**) Spectral RMS amplitude was 5.33 nm. (**C**) AFM tapping frequency.

Imaging of the printed graphene patterns on PI was performed using a SEM with a 2–5 kV accelerating voltage. The thickness is shown in Figure 5 that displays images of prints after annealing. AFM was applied to the graphene samples to characterize their thickness (Figure 6). A drop of graphene ink was positioned on a silicon slide and was dried on a warm plate in vacuumed chamber to ensure the precision of experiments.

### 2.8. Chip Biocompatibility Tests

**Figure 7:**
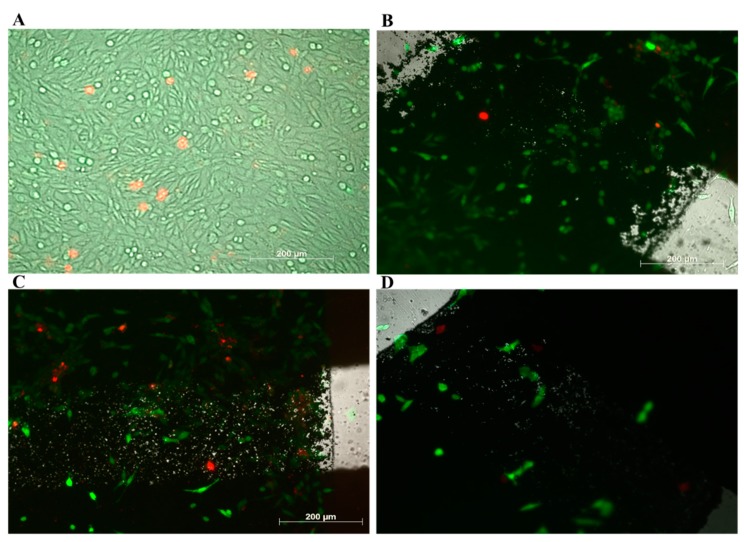
Cells cultured on graphene chips after 72 h of incubation. (**A**) Control well of N27 cells after 72 h at 37 °C in a 5% CO_2_ atmosphere. (**B**–**D**) After 72 h of incubation, the cells expanded across a gap with a length of 290 µm.

A flask of eight-time-passaged N27 cells was chosen for testing the biocompatibility of the graphene sensor. The graphene chips were placed in six-well cell culture plates. The chips were then placed under a biological safety cabinet and exposed to UV radiation for 12 h. The UV exposure ensured that the chips were sterile and biocompatibility would be maximized. In this study, N27s cells were cultured in 3 mL of maintenance medium (MM) that included RPMI medium 1640 (1X), 10% FBS, 1% penicillin, and 1% L-glutamine. Then, 3 mL medium was introduced in each well which was covered with the graphene chip. N27 cells with a cell density ≥1 × 10^6^ cells/vial were added to the chips using a micro pipette; 10 µL of cell suspension was introduced in each of the prints. The cells were left to grow in an incubator maintained at 37 °C with 5% CO_2_. The cells were checked under an inverted microscope at 72 h, as shown in Figure 7.

### 2.9. Live–Dead Cell Assay

Live–dead cell assays were performed using a 70 µM CellTracker™ CMFDA solution combined with an 8 µM propidium iodide (PI) solution in FBS-free RPMI medium. The CellTracker™ CMFDA solution was prepared by dissolving 50 µg of CellTracker™ in 10.8 µL of dimethyl sulfoxide (DMSO). The PI was diluted from a stock provided by Invitrogen. The 70 µM CellTracker™ CMFDA solution and 80 µM PI solution were dissolved in FBS-free RPMI medium to reach the correct concentrations. Then, 1 mL of solution was prepared to facilitate the calculations and minimize the error. MM was carefully removed from the desired well of the 6-well plate. After removal, the well was rinsed with FBS-free RPMI medium (500 µL), 500 µL of dye was added to the well, and the cells were then incubated for 30 min at 37 °C in a 5% CO_2_ atmosphere. The dye was then removed, and FBS-free RPMI medium was added to maintain moisture for sample imaging. N27 cells were imaged after 72 h of incubation. As shown in the control well (Figure 7A), live cells were colored in green, and dead cells were colored in red. Live and dead cells were also visible on the graphene chips. The chips were removed from the MM after incubation to ensure the cells were indeed growing on the chips and not on the well-plate surface, since the cells might have grown underneath the chips rather than on the chips. The chips were removed from the wells and transferred to clean wells with no MM.

A Zeiss Axio Observer Z1 inverted microscope was used to gather the live–dead cell assay results. The cells were carefully shielded from light after performing the live–dead cell assay so as to not affect the results. CellTracker™ CMFDA has an excitation of 492 nm and an emission of 517 nm. Propidium iodide has an excitation of 535 nm and an emission of 617 nm. The microscope was set to capture these wavelengths and image the fluorescence resulting from the live–dead cell assay. The results can be seen in Figure 7.

## 3. Results and Discussion

### 3.1. Chip Design

Traditional graphene-based biosensors are expensive and are still in a conceptual stage. One fabrication method consists in synthesizing graphene on a glass slip and etching SiO_2_ with a Si/285 nm laser (34). Another fabrication method uses surface plasmon resonance to fabricate graphene on a gold sensor to achieve higher sensitivity than that of a traditional gold thin film SPR sensor [45].

In our design, the graphene biosensor chip included three components: a glass coverslip, a copper tape, and a polyimide (Kapton) polymer substrate (Figure 1). The copper tape was fixed on both sides of the chip with a 0.5 mm gap and fully covered by Kapton PI (Figure 1B) to prevent any cellular exposure to copper. The chip may cause cytotoxic effects, and the goal was to minimize these effects [46]. Since the gap between the two pieces of copper tape was ≤0.5 mm, the Kapton PI substrate was used to connect the two pieces of tape with a flat surface. The effect of this gap on the 3D prints was negligible (Figure 1E) [47,48,49]. This design ensured that light could penetrate through the gap between the copper tape layers and reduced damage to the prints, offering an inexpensive physical method that is ready to use.

### 3.2. Printing Processes and Microscopy Studies

Aqueous graphene was pre-prepared using the wet-ball milling technology as described previously [50]. This paper focuses on graphene printing and sensor preparation. Graphene ink was applied to Kapton PI, using the custom-designed electronic inkjet printer represented in Figure 1. Because the FLG ink is hydrophilic and negatively charged [51], the PI substrate required some surface modification before successful printing could take place. The substrate was separately submerged into two different wetting agents, PSS and PEI, producing a hydrophilic layer on PI. This process created a hydrophilic buffer layer and changed the substrate from hydrophobic to hydrophilic [52]. A review of the literature disclosed that traditional inkjet printing uses a commercial printer that does not support high-viscosity ink. This negatively affects the print quality [24,53]. In this setup, graphene ink was directly injected using a needle and printed on a PI substrate. An electric field (Figure 1E) helped the ink fuse to the Kapton PI substrate. We found that printing at a flow rate of 3 µL/min, using a needle with an inner diameter of 300 µm and a 3 kV voltage, produced stable, continuous graphene prints with equal width and thickness (Figure 5A). The rather high value of the electric field was necessary to fix the conductive ink on the substrate [54].

To validate graphene quality, Raman spectroscopy of a graphene drop on SiO_2_ was performed using a laser with a wavelength of 532 nm (Figure 4). A couple of key points can be taken from the Raman characterization. The acquired spectrum experienced a sharp G peak at ~1569.19 cm^−1^, a symmetrical 2D peak at ~2689.55 cm^−1^, and a D peak at ~1348.33 cm^−1^, indicating that we had indeed achieved few-layer graphene [55]. Furthermore, the calculated ${\left(I_{D}/I_{G}\right)}_{Graphene}$ of the blender-mixed graphene was 0.11, while that of the wet-ball milled graphene was found to be 0.16. To further characterize the printed graphene, a SEM was used to produce SEM images (Figure 5) that confirmed that the graphene prints had an average width of 868 µm with no voids. This confirmed the purity of our graphene prints and that the graphene would be safe for biological use [56]. Additionally, The AFM results indicate a consistent thickness for the isolated graphene flake, which further confirms the Raman Spectra. 

### 3.3. Post-Treatment of Graphene Prints

After undergoing the printing process, a thermal annealing process needed to be applied to complete the prints. This post-processing was necessary to prevent further disruption of the conductive network. The traditional solvents and surfactants used to perform graphite exfoliation can carry over into graphene production and may disrupt the conductive networks [21,44]. An oven was pre-heated to 280 °C to thermally treat the printed graphene. Graphene-printed chips were annealed for 30 min before being removed for conductivity measurements. Evidence shows that the properties of annealed graphene change as different annealing temperatures and times are applied [10,21,57,58,59]. The annealing process helps minimize FLG flake-to-substrate defects, improves print resistance, and cleans off any polymer contaminants still present on the graphene surface [60,61,62].

Multiple conductivity measurements were taken using a multimeter. Resistance of all five printed lines was measured across the gap for every 1 mm. From measuring the line resistance, a conductivity measurement approximately at 6800 $S\cdot m^{-1}$ was performed (Figure 3). By measuring in at different temperatures and different times, the changes in conductivity resulted negligible [63,64].

### 3.4. Biocompatibility Testing with N27 Cells

**Figure 8:**
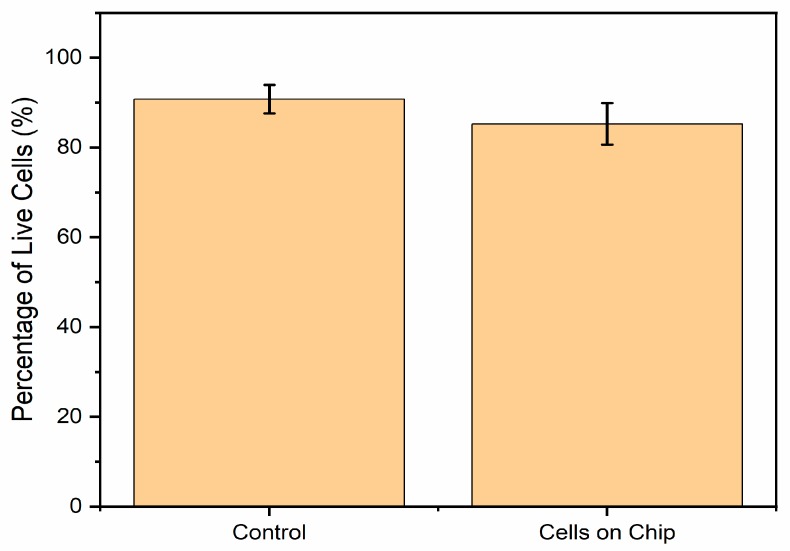
Percentage of live cells indicating that cell viability was approximately 85%.

First, 3 mL RPMI medium was added into each well, and a 50 µL cell sample was added into the medium. N27 cells were observed under an inverted microscope after incubation for 24, 48, and 96 h. Figure 8 indicates the growth rate of the cells, demonstrating that after 72 h, the cells accounted for 85% of the live screen area. A live–dead cell assay was performed to confirm the survival rate of the cells grown on graphene [65,66]. It was expected that the cells would grow on top of the annealed Kapton and graphene. Polyimide materials are often used in biosensor research because of their strength and broad compatibility across research areas [67]. N27 cells fully expanded across the gap, connecting both sides of the graphene prints (Figure 7) [68,69]. From the live–dead cell assay results, it appeared that N27 cells successfully grew on the graphene and Kapton substrate, confirming our earlier hypothesis [69,70].

It has been discussed in tissue-engineering literature that graphene may negatively affect the bimolecular mechanisms responsible for biological safety and toxicity [71,72,73,74]. Many reports have claimed that oxidative stress is one mechanism of cytotoxicity originating from carbon-based nanomaterials [75]. It is thought that graphene’s sharp edges may cause cell membrane damage by physical interaction and lead to cytotoxicity [75,76,77]. Studies have also found evidence that graphene oxide is less toxic than graphene, while reduced graphene oxide and hydrogenated graphene solutions are much more toxic to neural cells. This may be due to the larger flake sizes present in these samples [72,78,79,80,81]. Such evidence provides support for our setup described above, in which there was no detectable cytotoxicity to the cells. Optical images also showed that only a small proportion of the cells were affected at the locations studied. These results could be beneficial for future N27 cell electrophysiology studies.

## 4. Conclusions

In conclusion, an easily applied and inexpensive biosensor design was presented characterized by a high percentage (85%) of live cells. This design could maximize printed FLG conductivity up to 6800 $S\cdot m^{-1}$ after thermal annealing. On the basis of live–dead cell assays result, it also proved to be biocompatible and not to interfere with cell adhesion and neuronal cell proliferation. This design proved that pristine graphene after thermal treatment does not have a harmful effect on neuronal cells. In addition, graphene printed on Kapton PI exhibited no detectable adverse effects on cell multiplication, mitochondrial morphology, or cell stress (34). This highlights a promising future for graphene, including long-term and stable biomedical applications, especially in bioelectrical studies on N27 cell electrophysiology.
